# Risk Factors for the Occurrence of Cutaneous Neoplasms in Dogs: A Retrospective Study by Cytology Reports, 2019–2021

**DOI:** 10.3390/ani15142069

**Published:** 2025-07-14

**Authors:** Issa Carolina García-Reynoso, Cesar Augusto Flores-Dueñas, Nohemí Castro-del Campo, Mariana Jácome-Ibarra, José Carlomán Herrera-Ramírez, Sergio Daniel Gómez-Gómez, Miguel Ángel Rodríguez-Gaxiola, Soila Maribel Gaxiola-Camacho

**Affiliations:** 1 Institute for Research in Veterinary Sciences, Autonomous University of Baja California, Mexicali 21387, Mexico; issa.garcia@uabc.edu.mx (I.C.G.-R.); augusto.flores@uabc.edu.mx (C.A.F.-D.); mariana.jacome@uabc.edu.mx (M.J.-I.); jherrera20@uabc.edu.mx (J.C.H.-R.); gomez.sergio@uabc.edu.mx (S.D.G.-G.); 2 Faculty of Veterinary Medicine and Zootechnics, Autonomous University of Sinaloa, Culiacán 80260, Mexico; ncastro@uas.edu.mx (N.C.-d.C.); m_angel@uas.edu.mx (M.Á.R.-G.)

**Keywords:** canine cutaneous neoplasms, fine needle aspiration cytology, transmissible venereal tumor, mast cell tumor, risk factors, Mexico

## Abstract

Studies worldwide report cutaneous neoplasms in dogs; however, data in the arid regions of Mexico remain scarce. Here we report the main malignant cutaneous neoplasms diagnosed by fine needle aspiration cytology (FNAC), and describe the associations with age, sex and breed in Mexicali. Neoplastic lesions accounted for 25.52% (698/2735) of the cases, of which 56.59% (395/698) were malignant. The highest prevalence was observed in dogs aged 9–12 years (*n* = 193), intact males (*n* = 162), and mixed-breed dogs (*n* = 247). Round cell neoplasms (*n* = 309), including lymphoma, transmissible venereal tumors (TVT), and mast cell tumors (MCT), were the most common cell lineage. Using dogs aged 0–4 years as the reference group, dogs aged 9–12 years had 0.241 times the odds of developing malignant neoplasms (95% CI: 0.141–0.415, *p* = 0.0025). Using neutered males as the reference group, intact females showed 2.499 times the odds of developing malignant neoplasms (95% CI: 1.462–4.271, *p* = 0.0042). Compared to mixed-breed dogs, Schnauzers (OR = 0.161) showed significantly lower odds of malignancy (95% CI: 0.082–0.317, *p* = 0.0004), while Pitbull Terriers had 1.748 times more chance of present malignant neoplasia (95% CI: 1.014–3.013, *p* < 0.0001). This study provides significant epidemiological evidence on canine cutaneous neoplasms in an arid region of Mexico, identifying key risk factors and distribution patterns that can guide preventive, diagnostic, and therapeutic strategies tailored to regional characteristics.

## Simple Summary

Cutaneous neoplasms represent a significant health concern in dogs worldwide, yet epidemiological data from arid regions remain limited. This retrospective study analyzed 698 canine cutaneous neoplasms diagnosed via fine needle aspiration cytology in Mexicali, Mexico, where extreme desert conditions create unique environmental challenges. We found a notably high malignancy rate (56.59%), exceeding reports from temperate regions. Transmissible venereal tumor (TVT) and mast cell tumors comprised 28.35% and 27.84% of malignant cases, respectively. A paradoxical age distribution emerged, where young dogs (0–4 years) showed the highest malignancy rates, driven by endemic TVT affecting intact animals. Conversely, Schnauzers demonstrated protective effects against malignancy compared to mixed breeds. These findings reveal how regional factors (intense UV radiation, limited reproductive control, and local canine population management practices) create distinct cancer patterns in arid environments. Understanding these unique patterns is crucial for developing targeted prevention strategies, improving diagnostic approaches, and optimizing treatment protocols in settings where cytology frequently guides clinical decision-making.

## 1. Introduction

Cutaneous lesions of various origins are easily noticeable and therefore represent a common reason for veterinary consultation. Since many of these lesions share similar macroscopic characteristics and clinical signs [1], establishing a reliable diagnosis is crucial for providing accurate treatment and prognosis. Cutaneous neoplasms are the most frequently diagnosed type of cancer in dogs, making up approximately 30–40% of all canine neoplasms [2,3]. Their high prevalence, along with the diversity of clinical presentations and biological behaviors, poses a significant diagnostic and therapeutic challenge in veterinary practice [4].

Two primary diagnostic methods are frequently utilized: fine needle aspiration cytology (FNAC), a rapid, safe, cost-effective, and accessible technique with high sensitivity and specificity [5]; and histopathology, which, while regarded as the gold standard technique, tends to be more expensive, invasive, and requires a longer processing time. Recent studies have shown that FNAC has a sensitivity of 89.3–90.5% and specificity of 97.2–97.9% for diagnosing canine cutaneous neoplasms when performed by experienced pathologists [6,7]. It is important to note that worldwide, various factors, such as economic constraints and perceptions of invasiveness, limit the use of histopathological studies, with confirmation rates ranging from 15 to 25% in Latin America to 65–70% in developed countries [8,9]. In this context, FNAC has been established as a crucial diagnostic tool that provides valuable information to guide therapeutic decisions [10]. Furthermore, despite a lower number of cytological studies compared to histopathological papers, many publications exist on specific tumor types [11,12,13,14,15,16,17].

Among neoplasms in dogs, cutaneous neoplasms are the most common, with prevalence rates ranging from 33% [18] to 50% [19], although most of these neoplasms are benign [20]. The most frequently reported cutaneous neoplasms in dogs include mast cell tumors (MCTs), histiocytomas, cutaneous adnexal neoplasms, and lipomas [21]. The reported incidence varies considerably between geographic regions, suggesting that environmental, genetic, and population management factors may influence their development [22,23].

The epidemiology of canine cutaneous neoplasms has been extensively studied in developed countries. In the United States, MCTs account for 16–21% of all cutaneous neoplasms, followed by lipomas (8–10%), sebaceous gland adenomas (7–9%), and histiocytomas (4–8%) [24,25]. In Europe, similar patterns have been reported, although with significant regional variations [26,27]. Well-established risk factors include advanced age, certain breed predispositions (particularly in Boxers, Boston Terriers, and Retrievers for MCTs), and reproductive status [28,29].

The impact of environmental factors on the development of cutaneous neoplasms has gained considerable attention. Ultraviolet (UV) radiation exposure has been associated with an increased risk of squamous cell carcinomas, particularly in dogs with light-colored coats and in anatomical regions with lower hair density [30,31]. In arid and semi-arid areas, where UV radiation intensity is elevated throughout the year, this factor could have particular relevance [32]. Additionally, environmental pollutants, pesticides, and herbicides have been implicated in canine cutaneous carcinogenesis [33,34].

Although several studies have reported the frequency of cutaneous neoplasms in dogs worldwide, only a few have retrospectively analyzed large populations and identified risk factors associated with malignant cutaneous neoplasms [24,35,36,37,38,39,40]. In Latin America, epidemiological studies on canine cutaneous neoplasms are limited. Research in Brazil has reported a prevalence of malignant neoplasms of 40–45%, with transmissible venereal tumor (TVT), being particularly common due to high stray dog populations [41,42]. In Mexico, the few available studies have focused on central regions of the country, reporting similar patterns but with limited data on specific risk factors [43,44].

Identification of specific risk factors for malignant cutaneous neoplasms is crucial for developing preventive and early detection strategies. Recent molecular studies have identified mutations in genes such as c-KIT, BRAF, and p53 in MCTs and other cutaneous neoplasms, suggesting that certain breeds may have specific genetic predispositions [45,46]. Furthermore, the role of chronic inflammation, recurrent dermatitis, and non-healing wounds as predisposing factors for malignant transformation has been increasingly recognized [47,48].

However, data on the canine population in arid cities of Mexico remain scarce. The city of Mexicali, in the state of Baja California in Northwestern Mexico, presents unique environmental characteristics, including extreme temperatures (exceeding 45 °C in summer), low relative humidity, high UV radiation intensity throughout the year, and frequent dust storms [49]. These conditions could influence the epidemiology of cutaneous neoplasms differently from what has been reported in other regions. Additionally, local socioeconomic factors affecting access to preventive veterinary services and reproductive control could impact the prevalence of certain neoplasms such as TVT [50].

Therefore, the objective of this study was to report the main benign and malignant cutaneous neoplasms diagnosed through FNAC, in the domestic dog population of Mexicali, and to describe their frequency and associations with age, breed, and sex. This regional epidemiological knowledge is essential for guiding preventive, diagnostic, and therapeutic strategies adapted to the specific characteristics of the canine population in arid regions of Mexico.

## 2. Materials and Methods

This retrospective, cross-sectional study analyzed cytological records from 1 January 2019 to 31 December 2021. Data were obtained from two sources: the Veterinary Teaching Hospital of the Autonomous University of Baja California and a private reference clinical pathology laboratory, both located in Mexicali, Baja California, Mexico. Ethical approval was waived due to the retrospective nature of this study and the use of existing clinical records. All procedures complied with institutional guidelines.

**Figure 1:**
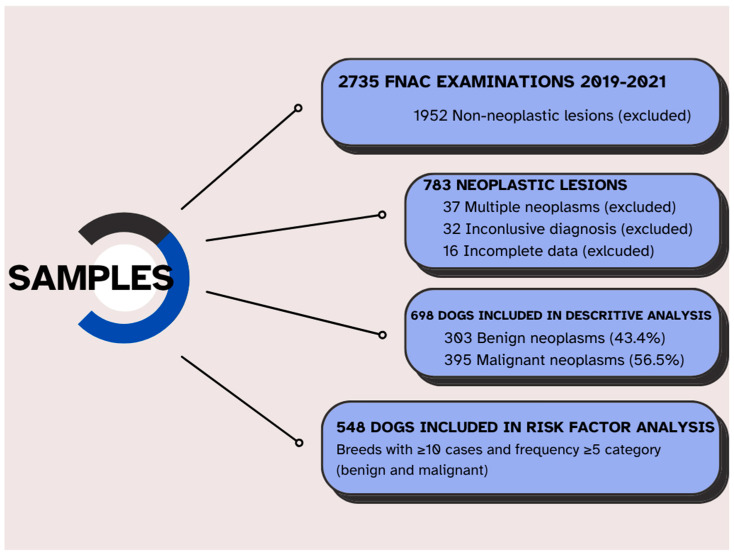
Flow diagram illustrating the total number of patients evaluated, those diagnosed with neoplasms, those with sufficient data for inclusion in the descriptive analysis, and those ultimately included in the risk factor analysis.

A total of 2735 fine-needle aspiration cytology (FNAC) studies were performed on dogs presenting cutaneous or subcutaneous nodules during the study period (817 in 2019, 757 in 2020, and 1161 in 2021). Dogs were eligible for inclusion if they had a single cutaneous or subcutaneous nodule evaluated by FNAC, a complete medical record including age, sex, reproductive status, breed, and a definitive cytological diagnosis. Cases were excluded if they had non-neoplastic lesions (*n* = 1952), inconclusive cytological results (*n* = 32), multiple distinct neoplasms (*n* = 37), or incomplete demographic data (*n* = 16). After applying these criteria, a total of 698 dogs with a single neoplastic lesion and complete records were included in the descriptive analysis. Subsequently, to perform the risk analysis study, we had to exclude 150 patients more, resulting in 548 individuals (Figure 1).

FNAC was performed using 22–23 gauge needles attached to 5–10 mL syringes. Multiple samples were collected from different areas of each lesion using non-aspiration techniques. Smears were air-dried and stained with modified Wright-Giemsa stain (Diff-Quik^®^, Siemens, Pomona, CA, USA), following the manufacturer’s instructions. All cytological samples were evaluated by a single board-certified veterinary clinical pathologist with over 10 years of experience. Neoplasms were classified and diagnosed cytologically according to the criteria described by Valenciano and Cowell [51]. Each lesion was categorized by biological behavior (benign or malignant), cell lineage (epithelial, mesenchymal, or round cell), and specific neoplasm type when cytological criteria were sufficient. To ensure diagnostic consistency, a random 10% of cases were re-evaluated, yielding an intra-observer agreement rate of 94%.

The following variables were recorded for each case: age (grouped into 0–4, 5–8, 9–12, and 13–18 years), sex and reproductive status (intact male, neutered male, intact female, spayed female), breed (as reported by owners and categorized per international breed standards), and neoplasm characteristics, including cytological diagnosis, and, when available, neoplasm location and size.

Descriptive statistics (frequencies and percentages) were calculated for all categorical variables. The association between malignancy and the variables of age, sex, and breed was assessed using the Wald chi-square test (χ^2^). A binary logistic regression was performed on breeds with more than 10 individuals and at least 5 individuals in every dependent variable (benign/malignant neoplasms), resulting in a dataset of 548 eligible dogs. Reference categories for multivariable analysis were obtained through stepwise backward selection, resulting in dogs aged 0–4 years, neutered males, and mixed-breed dogs. Odds ratios (OR) with 95% confidence intervals and *p*-values ≤ 0.05 were considered statistically significant. Statistical analyses were conducted using SAS (Statistical Analysis System) version 9.4.

## 3. Results

### 3.1. Descriptive Analysis

Over the three-year study period, 25.52% (698/2735) of cytological samples from cutaneous and subcutaneous masses were diagnosed as neoplastic lesions. Table 1 shows the frequency of breeds that presented at least one neoplasm. Of the 698 neoplastic cases, 303 (43.41%) were classified as benign, and 395 (56.59%) as malignant.

Dogs aged 9–12 years were most frequently affected (193/698, 27.65%), followed by those aged 5–8 years (187/698, 26.79%), 0–4 years (114/698, 16.33%), and 13–18 years (67/698, 9.59%). Regarding sex distribution, intact males represented the largest group (219/698, 31.37%), followed by intact females (187/698, 26.79%), spayed females (180/698, 25.78%), and neutered males (112/698, 16.04%). Mixed-breed dogs were most commonly affected (247/698, 35.38%), followed by Pitbull Terriers (94/698, 13.46%), Schnauzers (56/698, 8.02%), Chihuahuas (53/698, 7.59%), Poodles (38/698, 5.44%), and Labrador Retrievers (36, 5.15%). All remaining breeds represented 3.17% of the cases.

Classification by cell lineage revealed that round cell neoplasms were most prevalent (309/698, 44.26%), followed by mesenchymal (269/698, 38.53%) and epithelial neoplasms (120/698, 17.19%). Regarding the round cell neoplasms, 273/309 (88.34%) were malignant and 36/309 (11.65%) were benign. Mesenchymal neoplasms showed 78/269 (29.00%) malignant and 191/269 (71.00%) benign cases. Epithelial neoplasms comprised 44/120 (36.66%) malignant and 76/120 (63.33%) benign cases.

**Figure 2:**
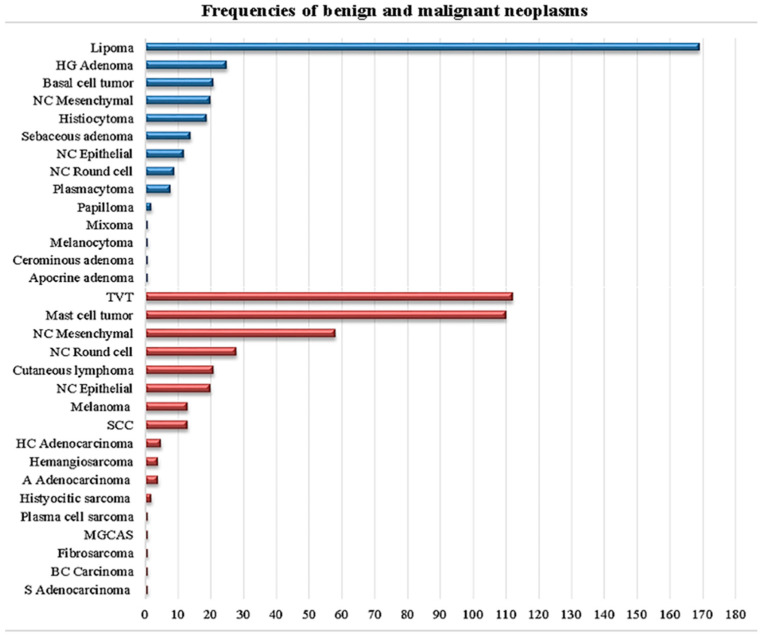
Benign (blue) and malignant (red) neoplasms and their frequencies. From top: HG = Hepatoid gland, NC = Not classified, TVT = Transmissible venereal tumor, SSC = Squamous cell carcinoma, HC = Hepatoid cell, A = Apocrine, MGCAS = Multinucleated giant cell anaplastic sarcoma, BC = basal cell, S = Sebaceous.

**Figure 3:**
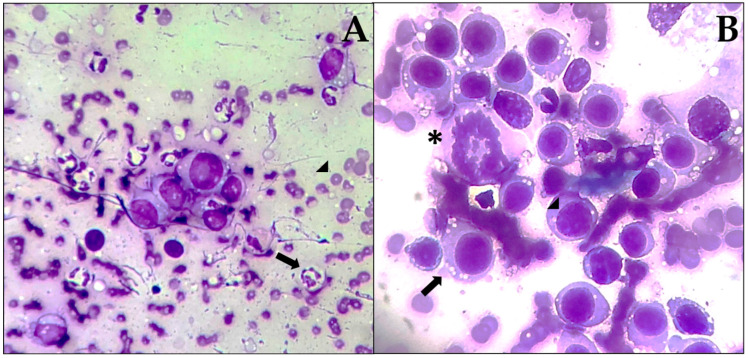
Diff-Quik^®^-stained slides from FNAC. (**A**) Samples with TVT typically show moderate to high cellularity, accompanied by abundant degenerated neutrophils (arrows) and erythrocytes (arrowheads) (20×). (**B**) Common cytological features of TVT include well-defined cytoplasmic borders with mild basophilia and prominent vacuolation (arrow); a large, round nucleus with coarse, granular chromatin; and one to three distinct nucleoli (arrowhead); anisocytosis, anisokaryosis, macrokaryosis, and occasional atypical mitotic figures (asterisk) are also observed (40×).

Figure 2 shows the frequency of the different types of neoplasms found, where the most frequently diagnosed were the following: lipoma (169/698, 24.21%), TVT (Figure 3) (112/698, 16.04%), MCT (110/698, 15.75%), unclassified malignant neoplasms (106/698, 15.18%), and unclassified benign neoplasms (41/698, 5.87%). Among the 303 benign neoplasms, the distribution was as follows: lipomas (169/303, 55.77%), hepatoid gland adenomas (25/303, 8.25%), basal cell neoplasms (21/303, 6.93%), unclassified mesenchymal neoplasms (20/303, 6.60%), histiocytomas (19/303, 6.27%), and nine additional neoplasm types representing the remaining 7.29%. Regarding the 395 malignant neoplasms, the distribution was: TVT (112/395, 28.35%), MCT (110/395, 27.84%), unclassified mesenchymal neoplasms (58/395, 14.68%), unclassified round cell neoplasms (28/395, 7.08%), cutaneous lymphoma (21/395, 5.31%), unclassified epithelial neoplasms (20/395, 5.06%), and 11 additional neoplasm types comprising the remaining 11.64%.

Age-specific analysis revealed distinct patterns for the most common neoplasms. Lipomas occurred most frequently in dogs aged 9–12 years (85/169, 50.29%) and 5–8 years (51/169, 30.17%). TVT showed the highest prevalence in dogs aged 0–4 years (67/112, 59.82%) and 5–8 years (33/112, 29.46%), with only 12/112 cases (10.71%) in dogs over 8 years old. MCT was most common in dogs aged 5–8 years (50/110, 45.45%) and 9–12 years (35/110, 31.81%).

Sex-specific patterns were evident for certain types of neoplasm. Hepatoid gland adenomas occurred predominantly in intact males (20/25, 80.00%), with only three cases in neutered males and two in intact females. TVT affected 58 intact males, 42 intact females, seven spayed females, and five neutered males, showing a clear predilection for intact animals (100/112, 89.29%). MCT showed a more even distribution across all sex categories, with no apparent predilection.

### 3.2. Risk Factors Analysis

Among breeds with at least ten individuals and more than four benign and malignant neoplasia, marked differences in malignancy rates were observed. Pitbull Terriers showed 72 malignant and 22 benign neoplasms (76.60% malignancy rate), as well as mixed breed dogs with 161 malignant and 86 benign neoplasms (65.18% malignancy rate). In contrast, Schnauzers demonstrated 13 malignant and 43 benign neoplasms (23.21% malignancy rate), while Poodles had 14 malignant and 24 benign neoplasms (36.84% malignancy rate) (Table 2). Although Dachshunds (*n* = 11) showed nine benign and only two malignant neoplasms, and Boxers (*n* = 21) presented exclusively with malignant neoplasms, including 18 MCT and three other malignant neoplasms, these breeds were selected as candidates for the risk factors study, given the statistical model constraints.

Multivariable logistic regression analysis of 548 dogs meeting the inclusion criteria identified significant predictors of malignancy (Table 3). Using dogs aged 0–4 years as the reference group, dogs aged 9–12 years demonstrated significantly lower odds (OR = 0.241, 95% CI = 0.141–0.415, *p* = 0.0025) of developing malignant neoplasia. Regarding sex and reproductive status, using neutered males as the reference group, intact females showed a 2.499-fold increase in the probability of developing malignant neoplasms (95% CI = 1.462–4.271, *p* = 0.0042). Breed analysis, compared to mixed-breed dogs, revealed significant protective effects for Schnauzers (OR = 0.161, 95% CI = 0.082–0.317, *p* = 0.0004) and a 1.748-fold increase in the probability of presenting malignant neoplasms in Pitbull Terriers (95% CI = 1.014–3.013, *p* < 0.0001).

## 4. Discussion

Cutaneous neoplasms are among the most common neoplasms in dogs, yet epidemiological studies in arid regions of Latin America remain scarce. This study offers key insights into the distribution of cutaneous neoplasms in the canine population of Northwestern Mexico, enabling comparisons with global data and contributing to the identification of specific risk factors that may guide preventive and diagnostic strategies in this region.

The higher proportion of malignant neoplasms (56.59%) observed in this study exceeds the prevalence reported in European (45.2%) [26] and North American (approximately 50%) studies [24], suggesting possible environmental and clinical management differences in the studied region. This elevated malignancy rate likely reflects the intense UV radiation exposure characteristic of Mexicali’s desert environment, where annual UV index values frequently exceed 11 (extreme category). Supporting this environmental hypothesis, studies from similarly high-UV regions demonstrate parallel patterns: Grenada reports hemangiosarcoma comprising 19.1% of cutaneous neoplasms [52], while Brazilian studies document that 27–80% of canine hemangiosarcomas are cutaneous in high-UV areas compared to only 14% in North America [53]. The molecular signature of UV-induced damage, TP53 mutations in 59.6% of hemangiosarcoma cases, and PIK3CA pathway alterations [54], provide mechanistic support for solar radiation as a primary driver of cutaneous malignancy in our region.

Statistical analysis revealed unexpected protective factors against malignant neoplasms. Using dogs aged 0–4 years as the reference group, the 9–12-year-old group demonstrated significantly lower odds of developing malignant neoplasms (OR = 0.241, 95% CI = 0.141–0.415, *p* < 0.0025). This finding appears paradoxical given the established principle of age-related cancer accumulation; however, the explanation lies, apparently, in the specific neoplastic distribution within our population. The exceptionally high prevalence of TVT in young dogs (59.82% of TVT cases occurred in dogs aged 0–4 years) creates an artificial peak in malignancy rates that obscures typical age-related cancer progression. When considering non-transmissible neoplasms alone, the expected pattern emerges, consistent with the Swiss Cancer Registry data, showing peak incidence rate ratios in 8–11-year-old dogs (IRR: 18.2) [55]. This finding underscores the importance of considering regional disease patterns when interpreting epidemiological data, as endemic diseases like TVT can significantly alter apparent risk factor associations.

Analysis by cell lineage revealed intrinsic biological patterns: round cell neoplasms were predominantly malignant (88.34%), whereas mesenchymal neoplasms were mostly benign (71.00%), consistent with molecular mechanisms documented in previous studies [40,41]. Round cell neoplasms, including lymphoma, mast cell tumors, and TVT, derive from mobile cells of the hematopoietic and immune systems whose inherent capacity for migration predisposes them to aggressive behavior. Recent genomic analyses reveal that these neoplasms frequently harbor mutations in genes associated with increased invasiveness and metastatic potential, including GNB1 mutations in 17.3% of mast cell tumors [56]. Conversely, mesenchymal neoplasms arising from connective tissues, predominantly lipomas in our study, typically maintain the differentiation and growth constraints of their cells of origin.

Among malignant neoplasms, TVT and MCT were the most frequent, representing 28.35% and 27.84% of cases, respectively. The high incidence of TVT in our study reinforces the well-established link between reproductive control and disease distribution. In countries like the United States and parts of Europe, where early spaying and neutering are common, TVT prevalence is significantly lower [42]. Similar TVT prevalence rates have been reported in studies from Brazil [9] and Mexico [43], confirming its status as a significant veterinary public health concern in regions with limited reproductive control measures. The predominance of TVT in animals younger than 9 years old (89.28%) emphasizes the importance of implementing comprehensive reproductive control programs as a primary prevention strategy.

MCT prevalence in our study aligns with global reports [24], confirming its status as one of the most common cutaneous neoplasms in dogs. In a large German epidemiological study analyzing 109,616 histopathological samples, MCT represented 9.7% of all canine neoplasms, ranking as the third most frequent neoplasm type [57]. Importantly, recent advances in cytological evaluation have demonstrated that FNAC can provide valuable prognostic information for MCT. Using specific cytological criteria, high-grade MCTs can be identified with 88% sensitivity and 94% specificity, with dogs having high-grade neoplasms being 25 times more likely to die within 2 years [13]. This prognostic capability positions cytology as a valuable decision-making tool that can guide initial clinical management, inform owners about prognosis, and help prioritize cases for surgical intervention or referral while treatment options are being evaluated.

Our findings regarding sex and reproductive status revealed that intact females had significantly higher odds (OR = 2.499) of developing malignant neoplasms compared to neutered males. This observation contrasts with numerous studies reporting increased cancer risk associated with neutering. For instance, the Veterinary Medical Database analysis indicated that spayed females had a 72% higher risk of developing hemangiosarcoma (OR = 1.72); meanwhile, neutered males exhibited a 14% increased risk (OR = 1.14) compared to intact animals [58]. Additionally, Vizslas demonstrated a 3.5-fold higher risk of MCTs following neutering [59]. Moreover, data from the Swiss Canine Cancer Registry (2008–2020) also indicated a higher proportion of skin neoplasms (30.01%) in neutered females [55].

However, the existing literature presents variable results depending on population characteristics. For example, a VetCompass study involving 168,636 dogs reported significantly reduced odds for MCT in neutered dogs (OR = 0.1, 95% CI: 0.1–0.2) [60], these findings are more consistent with ours. Such contradictory outcomes likely stem from regional differences: areas with high neutering prevalence, such as the USA (85%), display distinct cancer profiles compared to regions with predominantly intact populations, like Sweden (99%) [61].

The key distinction in our population is the notably high prevalence of transmissible venereal tumors (TVT), which represent 28.35% of malignant neoplasms, predominantly affecting young, intact animals (89.28%). In populations with low neutering rates, where TVT has a significant influence on neoplasm epidemiology, a protective effect may appear more pronounced in neutered animals. Moreover, in Mexico, neutering often correlates with improved veterinary care and reduced roaming behaviors, factors inherently linked to decreased TVT exposure [42,50].

Due to the lack of data on the age at neutering, our study was unable to assess the documented timing-related effects. For example, early neutering (before 12 months) in Golden Retrievers is associated with a higher incidence of lymphoma (10% vs. 3% in intact males), while late-neutered females show a higher incidence of MCT (5.7% vs. 0% in intact females) [62]. These breed- and timing-specific nuances were beyond the analytical scope of our study.

For hepatoid gland adenoma, we found that only 3.58% of the study population presented it, in contrast to studies that report frequencies of 7–25% [24,63]. Regarding sex, we found a male: female relationship of 23:2, which is very similar to Petterino et al., (2004) [64] where they report a 20:4 male:female relation, including five carcinomas among these 24 neoplasms, and adding the relevance of growing hormone-dependence in the presence of this adenoma, which is beyond the scope of this paper.

Breed analysis revealed significant protective effects for Schnauzers (OR = 0.161) compared to mixed-breed dogs, contradicting common assumptions about purebred cancer predisposition. This finding appears paradoxical given documented genetic risk factors in these breeds: Giant and Standard Schnauzers carry elevated KITLG gene copy number variations, which may be associated with digital squamous cell carcinoma [65].

For Pitbull Terriers, there was a 1.784 times greater probability of developing malignant neoplasms compared to mixed-breed dogs, corroborating previous observations on genetic susceptibility [28]. Mutations in the c-KIT proto-oncogene, identified in the Pitbull Terrier among other breeds, lead to constitutive activation of the KIT receptor, promoting uncontrolled cell proliferation and increasing tumor aggressiveness [45,47], which explains the higher MCT prevalence observed in this breed in this study. Additionally, regional breeding populations may harbor different genetic variants; regional legislations as well as regional culture may impact the number of dogs of this breed present in Mexico, Latin America, North America, or Europe. Furthermore, breed-specific characteristics such as coat density may confer protection against UV radiation in desert regions; thus, UV radiation in cutaneous carcinogenesis appears particularly relevant in Mexicali’s arid environment. Geographic epidemiology demonstrates clear associations between desert climates and specific neoplasm types, with Australian research showing 15% higher UV radiation exposure compared to equivalent northern latitudes [66]. UV-associated neoplasms show distinct patterns: cutaneous hemangiosarcoma correlates strongly with solar exposure, particularly affecting the ventral abdomen and sparsely haired areas in light-coated breeds [67]. The molecular evidence supporting UV-induced carcinogenesis, including characteristic alterations in TP53 mutations and PIK3CA pathway [54], suggests that environmental factors significantly contribute to the elevated malignancy rate observed in our population.

Although FNAC achieved complete classification in 94.13% of benign and 84.82% of malignant neoplasms, important limitations must be acknowledged. The technique cannot reliably differentiate morphologically similar round cell neoplasms without the use of ancillary methods, although in our high-TVT endemic region (Figure 3), the clinical context (young, intact animals, and genital/facial location) aids in diagnosis. Critical prognostic features, such as surgical margins, histologic grade (Patnaik/Kiupel), or lymphovascular invasion, cannot be assessed cytologically. Mesenchymal neoplasms, which comprise 38.53% of our cases, present particular diagnostic challenges as cytology cannot differentiate specific sarcoma subtypes. This limitation contributed to our overall incomplete classification rates. While histopathology remains the gold standard for definitive neoplasm diagnosis, economic constraints significantly limit its use in Latin American veterinary practice, with confirmation rates ranging from only 15 to 25% compared to 65–70% in developed countries [8,9]. This economic reality makes FNAC particularly relevant in our regional context, as it provides clinically actionable information at a fraction of the cost of histopathological examination. Despite its limitations, FNAC achieved complete classification in 78.93% of overall samples in our study, enabling appropriate clinical decision-making for the majority of patients. The high concordance between cytological and histopathological diagnoses, as reported in validation studies (89–90% sensitivity and 97% specificity) [6,7], supports FNAC as a valuable, though not infallible, first-line diagnostic tool when used by experienced pathologists. This is particularly crucial in settings where financial constraints preclude routine histopathological confirmation. This study thus reflects real-world veterinary practice in resource-limited regions, where treatment decisions must often rely on cytological findings, emphasizing the importance of understanding both the capabilities and limitations of FNAC when interpreting epidemiological patterns of cutaneous neoplasms.

This study has several limitations, including its retrospective nature and the use of convenience sampling, which may introduce bias in the prevalence estimates. Environmental factors such as specific UV exposure levels, coat color, lifestyle factors, and age at neutering were not systematically evaluated. The inability to confirm all cytological diagnoses histologically may affect accuracy; however, recent validation studies support the reliability of FNAC for most cutaneous neoplasms [68,69,70]. Despite these limitations, this study represents a significant contribution to the epidemiology of cutaneous neoplasms in arid regions of Latin America, revealing unexpected protective factors and risk patterns that challenge established paradigms and warrant further investigation.

## 5. Conclusions

This comprehensive analysis of 698 canine cutaneous neoplasms from an arid region of Mexico reveals that regional factors greatly influence cancer epidemiology. The high prevalence of malignant neoplasms likely reflects the intense UV radiation exposure typical of desert environments. The apparent increased risk in younger dogs is mainly attributable to endemic TVT, emphasizing the urgent need for reproductive control programs. Unexpected increased risk in intact females and Schnauzers suggests complex interactions between genetic, environmental, and socioeconomic factors that require further investigation. These findings underscore the importance of developing region-specific preventive strategies that consider local disease patterns, environmental conditions, and population characteristics rather than extrapolating from studies conducted in disparate geographic and socioeconomic contexts. Future prospective studies incorporating environmental monitoring, genetic profiling, and molecular characterization will further elucidate the complex interplay between genetic susceptibility and environmental carcinogens in canine cutaneous oncogenesis.

## Figures and Tables

**Table 1:** Dogs with complete data and one type of neoplasm.

Breed	Freq.	Breed	Freq.	Breed	Freq.
Akita	1	Fila Brasileiro	1	Poodle	38
Belgian Shepherd	3	French Bulldog	4	Pug	13
Boston Terrier	4	German Shepherd	14	Portuguese Water Dog	1
Bull Terrier	2	Golden Retriever	5	Rat Terrier	1
Basset Hound	7	Greyhound	1	Rottweiler	6
Beagle	7	Great Dane	4	Saint Bernard	2
Boxer	21	Jack Russell Terrier	2	Schnauzer	56
Chihuahua	53	Labrador Retriever	36	Shar Pei	3
Chow Chow	2	Malinois B. Shepherd	1	Shih Tzu	10
Cocker Spaniel	7	Maltese	6	Siberian Husky	9
Corgi	1	Miniature Pinscher	1	Springer Spaniel	2
Dachshund	11	Mixed-breed dogs	247	Swiss Shepherd	1
Doberman	4	Old English Sheepdog	1	Weimaraner	1
Dogo Argentino	2	Pekingese	1	Xoloitzcuintle	1
Dogue of Bordeaux	1	Pitbull Terrier	94	Yorkshire Terrier	4
English Bulldog	4	Pointer	2	Total	698

**Table 2:** Breeds with at least 10 individuals and a minimum of four benign and four malignant neoplasms were included in the chi-squared test. Boxers and Dachshunds were excluded from the analysis since they presented 3:18 and 9:2 benign: malignant neoplasms, respectively.

Breed	Benign	Malignant	Frequency (%)
Chihuahua	26	27	53 (9.67)
German Shepherd	7	7	14 (2.55)
Labrador Retriever	22	14	36 (6.56)
Mixed-breed dogs	86	161	247 (45.07)
Pitbull Terrier	22	72	94 (17.15)
Poodle	24	14	38 (6.93)
Schnauzer	43	13	56 (10.21)
Shih Tzu	5	5	10 (1.82)
Total	235	313	548

**Table 3:** Multivariable logistic regression analysis of risk factors for malignant neoplasia in dogs (*n* = 548).

Variable	n (%)	Adjusted Odds Ratio	95% CI	*p*-Value
Age group (years)				
0–4 years (reference)	99 (18.06)	1.00	—	—
5–8 years	181 (33.02)	0.614	0.353–1.067	0.1366
9–12 years	**193 (** **35.21)**	**0.241**	**0.141–0.415**	0.0025 *
13–18 years	75 (13.68)	0.295	0-155–0563	0.1256
Sex and Reproductive Status				
Neutered male (reference)	91 (16.60)	1.00	—	—
Intact female	**148 (27.00)**	**2.499**	**1.462–4.271**	0.0042 *
Spayed female	147 (26.82)	1.365	0.807–2.308	0.2492
Intact male	162 (29.56)	2.004	1.190–3.372	0.1387
Breed				
Mixed breed (reference)	247 (45.07)	1.00	—	—
Chihuahua	53 (9.67)	0.555	0.305–1.010	0.7705
Labrador Retriever	36 (6.56)	0.340	0.166–0.698	0.2079
German Sheperd	14 (2.55)	0.534	0.181–1.573	0.9304
American Pit Bull Terrier	**94 (17.15)**	**1.748**	**1.014–3.013**	<0.0001 *
Poodle	38 (6.93)	0.312	0.153–0.633	0.1217
Schnauzer	**56 (10.21)**	**0.161**	**0.082–0.317**	0.0004 *
Shih Tzu	10 (1.82)	0.534	0.150–1.896	0.9405

CI = confidence interval. Reference categories are indicated in italics. Statistical significance with bold and *.

## Data Availability

All data are available upon request.

## Abbreviations

The following abbreviations are used in this manuscript:

FNAC	Fine needle aspiration cytology
MCT	Mast cell tumors
TVT	Transmissible venereal tumor
OR	Odds ratio
CI	Confidence intervals
